# *CDKN1C* (p57^KIP2^) Is a Direct Target of EZH2 and Suppressed by Multiple Epigenetic Mechanisms in Breast Cancer Cells

**DOI:** 10.1371/journal.pone.0005011

**Published:** 2009-04-02

**Authors:** Xiaojing Yang, R. K. Murthy Karuturi, Feng Sun, Meiyee Aau, Kun Yu, Rongguang Shao, Lance D. Miller, Patrick Boon Ooi Tan, Qiang Yu

**Affiliations:** 1 Cancer Biology and Pharmacology, Genome Institute of Singapore, A*STAR (Agency for Science, Technology and Research), Biopolis, Singapore; 2 Institute of Medicinal Biotechnology, Chinese Academy of Medical Sciences, Beijing, China; 3 Information and Mathematical Science, Genome Institute of Singapore, A*STAR (Agency for Science, Technology and Research), Biopolis, Singapore; 4 Department of Pharmacy, National University of Singapore, Singapore, Singapore; 5 Duke-NUS Graduate Medical School, Singapore, Singapore; 6 Cell and Medical Biology, Genome Institute of Singapore, A*STAR (Agency for Science, Technology and Research), Biopolis, Singapore; Ordway Research Institute, United States of America

## Abstract

*CDKN1C* (encoding tumor suppressor p57^KIP2^) is a cyclin-dependent kinase (CDK) inhibitor whose family members are often transcriptionally downregulated in human cancer via promoter DNA methylation. In this study, we show that *CDKN1C* is repressed in breast cancer cells mainly through histone modifications. In particular, we show that *CDKN1C* is targeted by histone methyltransferase EZH2-mediated histone H3 lysine 27 trimethylation (H3K27me3), and can be strongly activated by inhibition of EZH2 in synergy with histone deacetylase inhibitor. Consistent with the overexpression of *EZH2* in a variety of human cancers including breast cancer, *CDKN1C* in these cancers is downregulated, and breast tumors expressing low levels of *CDKN1C* are associated with a poor prognosis. We further show that assessing both *EZH2* and *CDKN1C* expression levels as a measurement of EZH2 pathway activity provides a more predictive power of disease outcome than that achieved with *EZH2* or *CDKN1C* alone. Taken together, our study reveals a novel epigenetic mechanism governing *CDKN1C* repression in breast cancer. Importantly, as a newly identified EZH2 target with prognostic value, it has implications in patient stratification for cancer therapeutic targeting EZH2-mediated gene repression.

## Introduction

Cyclin-dependent kinase inhibitors (CDKIs) are a large family of proteins that regulate cell cycle progression, cell proliferation and differentiation. CDKIs show widespread involvement in tumor suppression and are deregulated in many types of human cancers by genetic and epigenetic alterations. Loss of expression of CDKIs, such as p16^INK4A^, due to promoter DNA hypermethylation, is frequent in human cancer and the CDKIs downregulation is associated with aberrant cell proliferation and tumor growth. In addition to promoter DNA methylation, histone modifications also play a role in inactivation of the CDK inhibitors. It has been shown that H3K9 methylation can occur at p16^INK4A^ independently of DNA methylation [1]. More recently, inactivation of the INK4A locus by other mechanisms such as Polycomb-associated histone H3K27 methylation have also been reported [2], [3], [4].

Pharmacological reagents have been used to understand the regulatory components of gene silencing in cancer. DNA methylation inhibitor 5-Aza-2′-deoxycytidine (5-Aza-C) has been used extensively to restore the expression of genes silenced by DNA methylation [5], whereas histone deacetylase (HDAC) inhibitors such as Trichostatin A (TSA) can induce gene expression by reversing repressed chromatin [6]. These two classes of agents can also act in synergy for the reactivation of epigenetically silenced genes [7]. In addition to histone deacetylation, histone methylation also contributes to gene silencing. In particular, Polycomb protein EZH2 (Enhancer of Zeste 2) is a histone methyltransferase that is often overexpressed in human cancers and is associated with cancer aggressiveness [8], [9]. EZH2 specifically methylates lysine 27 of histone H3 (H3K27), a repressive chromatin mark associated with gene silencing [10], [11], [12] and often represses target genes associated with growth control. It has been shown that EZH2-mediated gene repression requires HDAC activity [13] and its functional relationship to DNA methylation is also of current interest [14], [15], [16], [17], [18]. Furthermore, recent studies indicate that inhibiting DNA methylation alone or even with the aid of HDAC inhibitors is insufficient to induce an euchromatic chromatin state due to the retention of repressive histone marks [19], [20]. These findings suggest that for a stable reversion of epigenetic silencing in cancer, a complete reversal from the malignant heterochromatin to a normal euchromatin is required.

CDK inhibitor *CDKN1C* (p57^KIP2^) has been previously reported to be inactivated via promoter DNA methylation in a variety of human cancers [21], [22]. In this study, we report that the *CDKN1C* is repressed in breast cancer by multiple epigenetic mechanisms. We demonstrate that the *CDKN1C* gene is the target of EZH2-mediaed trimethylation of histone H3 at lysine 27 (H3K27me3) that coordinates with histone deacetylation to suppress *CDKN1C* expression. Combined treatment of DZNep, an inhibitor of histone methylation [23], with HDAC inhibitor TSA causes a robust reactivation of *CDKN1C* expression. We also demonstrate the prognostic value of EZH2-mediated *CDKN1C* repression in breast cancer and suggest its clinical significance for EZH2-targeted cancer therapeutics.

## Results

### *CDKN1C* repression in breast cancer cells is associated with histone modifications independently of DNA methylation

We have previously reported that S-adenosylhomocysteine hydrolase inhibitor 3-Deazaneplanosin A (DZNep) is able to inhibit histone methylation and depletes the EZH2 complex and the associated H2K27 methylation [23] and combination of DZNep with HDAC inhibitor TSA resulted in robust activation of H3K27me3 target genes [16]. In an effort to comprehensively understand the epigenetic events in breast cancer, we performed gene expression analysis in various breast cancer cell lines treated with DZNep, TSA or Aza, alone or in various combinations. We found that *CDKN1C* can be strongly induced by a combination of DZNep and TSA treatment compared to a modest induction by Aza (see below). This finding suggests that *CDKN1C* expression in breast cancer is predominately regulated by histone modifications instead of DNA hypermethylation.

**Figure 1:**
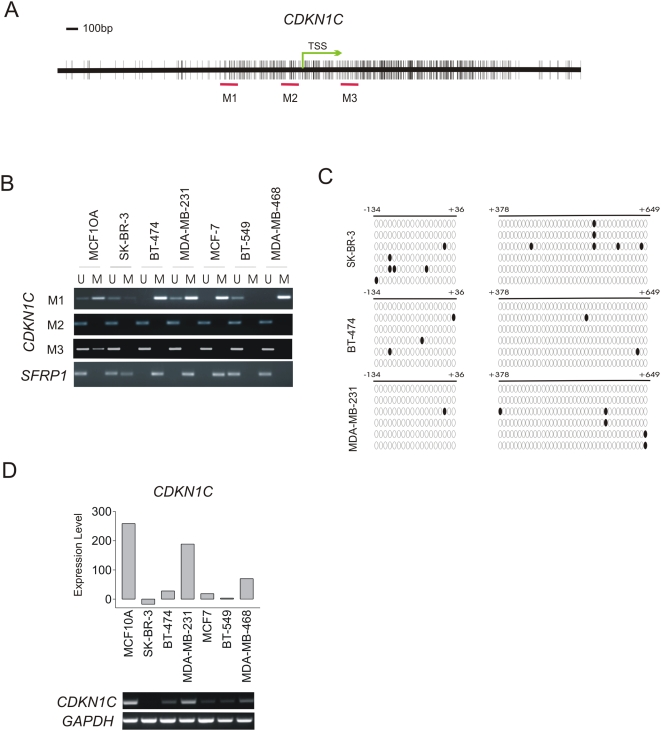
DNA methylation status of *CDKN1C* promoter in breast cancer cells. (A) Schematic representation of *CDKN1C* locus. Vertical bars indicate the CpG sites. TSS, transcription start site. M1, M2 and M3 represent genomic regions for methylation specific PCR (MSP) analysis. (B) MSP analysis of genomic regions surrounding the TSS of *CDKN1C*. *SFRP1* was used as a positive control of effective bisulfite conversation. (C) Bisulfite genomic DNA sequencing results of indicated regions in SK-BR-3, BT-474 and MDA-MB-231 cells. (D) Upper panel, expression values of *CDKN1C* in Illumina expression data of breast cancer cell lines. Lower panel, expression of *CDKN1C* in indicated cell lines are determined by RT-PCR.

To validate this finding, we first examined the relationship between the DNA methylation status of the *CDKN1C* promoter and the levels of *CDKN1C* expression in various breast cancer cell lines as well as the non-cancerous breast epithelial cell line (MCF10A). To this end, we have used methylation-specific PCR (MSP) to examine three regions flanking the entire CpG island surrounding the transcription start site (TSS) (Figure 1A). We found that the immediate promoter regions (M2 and M3) appeared to be unmethylated in all the breast cancer cell lines tested (Figure 1B), though the similar regions have been previously found to be methylated in lung cancer [21]. In a more distal region (M1) 500 bp upstream of the TSS, DNA was found to methylated in certain breast cancer cell lines, including BT-474 and MDA-MB-231, as well as in MCF10A cells, indicating this methylation is not cancer specific (Figure 1B). Bisulfite genomic sequencing results further confirmed the lack of methylation in M2 and M3 regions in SK-BR-3, BT-474 and MDA-MB-231 cells (Figure 1C). Examination of *CDKN1C* expression from our breast cancer cell lines gene expression database indicates that *CDKN1C* expression displayed varied levels of expression but in general was reduced in most of the breast cancer cell lines (except the MDA-MB-231 cells) as compared to MCF10A cells (Figure 1D). RT-PCR analysis further validated the array data (Figure 1D). Of important notice, the expression pattern of *CDKN1C* does not seem to correlate with the methylation status in these cell lines.

**Figure 2:**
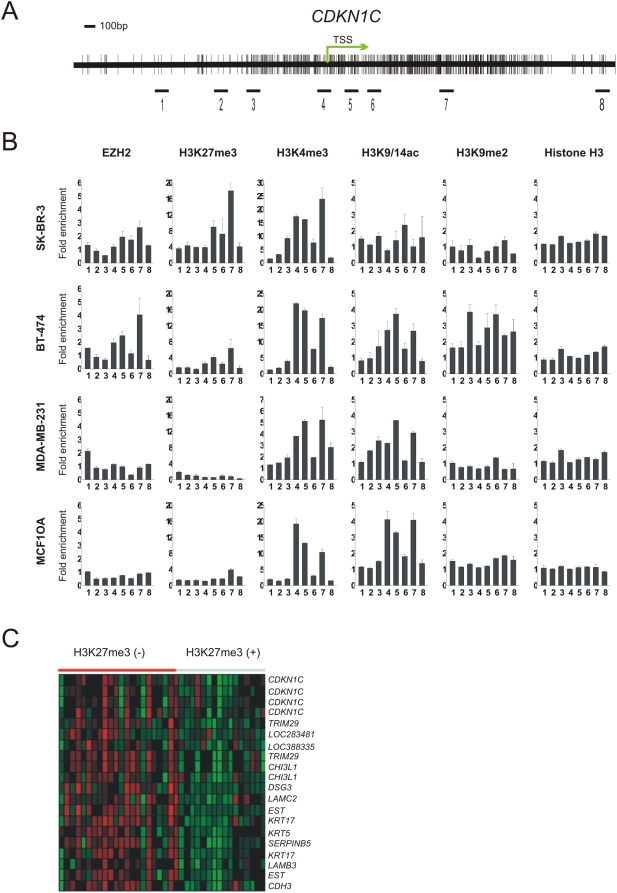
Histone modifications at *CDKN1C* locus in breast cancer cells. (A) Schematic representation of *CDKN1C* locus, TSS (transcription start site). Numbered bars indicate the genomic regions analyzed for histone modifications using Chromatin immunoprecipitation (ChIP) assay. (B) ChIP assay analysis of indicated histone marks and EZH2 binding at *CDKN1C* locus in SK-BR-3, BT-474 and MDA-MB-231 breast cancer cells, as well as none-cancerous epithelial breast MCF10A cells. The enrichments of examined histone marks in the indicated regions were examined by quantitative PCR relative to input DNA. Error bars were calculated as standard error (±s.d.). (C) Gene cluster showing the differential expression of *CDKN1C* and correlated genes in H3K27me3 positive and negative breast cancer samples. The data was obtained through analyzing a breast cancer microarray dataset in Oncomine database (www.oncomine.org).

We next set out to determine whether histone modifications are responsible for *CDKN1C* repression in breast cancer. We used chromatin immunoprecipitation (ChIP) coupled with quantitative PCR to assess the following histone marks: the repressive chromatin marks H3K27me3, H3K9me3, H3K9me2, and H4K20me3, as well as activating H3K4me3 and acetylated histone (H3K9/14 ac) that are normally associated with gene activation. A series of PCR primer sets were designed to probe the 4 kb chromatin region surrounding the *CDKN1C* TSS (Figure 2A). We comprehensively characterized the chromatin state in SK-BR-3, BT-474, MDA-MB-231 and MCF10A cell lines in which *CDKN1C* is expressed at different levels. We detected an abundant H3K27me3 in a region approximately 300 bp downstream of the TSS in SK-BR-3 cells that express the lowest level of *CDKN1C* (Figure 2B). This was also observed to a lesser extend in BT-474 cells that express a modest level of *CDKN1C*, and at low rates in MDA-MB-231 and MCF10A cells that express abundant *CDKN1C*. Consistent with the enrichment of H3K27me3, we also detected a strong binding of H3K27 methyltransferase EZH2 to the *CDKN1C* promoter in SK-BR-3, to a lesser extend in BT-474 cells but not in MDA-MB-231 and MCF10A cells. Thus, levels of enrichment of EZH2 and H3K27me3 correlate very well (inversely) with the levels of *CDKN1C* expression across these diverse cell lines. This finding is consistent with the notion that EZH2 and the associated H3K27me3 enrichment are correlated with gene repression in cancer cells, and the majority of H3K27me3 is detected in the region downstream of the TSS [24], [25], [26], [27].

In addition, abundant H3K4me3 near the TSS was detected in all the four cell lines, irrespective of the levels of *CDKN1C* expression (Figure 2B). This suggests that *CDKN1C* is marked by both repressive and activating histone marks in SK-BR-3 and BT-474 cells; a bivalent chromatin state that is generally associated with gene repression [28], [29]. Higher abundance of acetylated H3 (H3K9/14ac) was detected in *CDKN1C*-expressing MCF10A, MDA-MB-231 and BT-474 cells but was less detectable in SK-BR3 cells that express a lowest level of *CDKN1C*, indicating a positive correlation of H3K9/14ac with *CDKN1C* expression in these cells. We also detected the presence of another repressive mark H3K9me2 in BT-474 cells but not in other three cell lines (Figure 2B). Taken together, these results indicate that the chromatin states reflecting the abundance of repressive H3K27me3; activating H3K4me3 and H3K9/14ac, as well as their combinatorial effects, correlate well with the levels of *CDKN1C* expression in these cells. Other repressive histone modifications such as H4K20me3 and H3K9me3 were not detected at the *CDKN1C* locus (data not shown).

To further determine the association of H3K27me3 with the level of *CDKN1C* expression in human breast tumors, we took the advantage of an independent microarray dataset of human breast tumors stained with H3K27me3 in Oncomine microarray database (www.oncomine.org). Figure 2C shows that breast tumors stained positive for H3K27me3 display a consistent downregulation of *CDKN1C* compared with those negative for H3K27me3. Of important note, genes exhibiting the similar expression patterns to *CDKN1C* include *KRT17*, *KRT5* and *LAMB3* that have been validated to be EZH2 target in our previous study [23]. Thus, the data from both clinical breast tumor samples and cancer cell lines all support that *CDKN1C* downregulation in breast cancer is associated with a higher level of H3K27me3.

### Robust activation of *CDKN1C* expression by a combination treatment with DZNep and TSA

**Figure 3:**
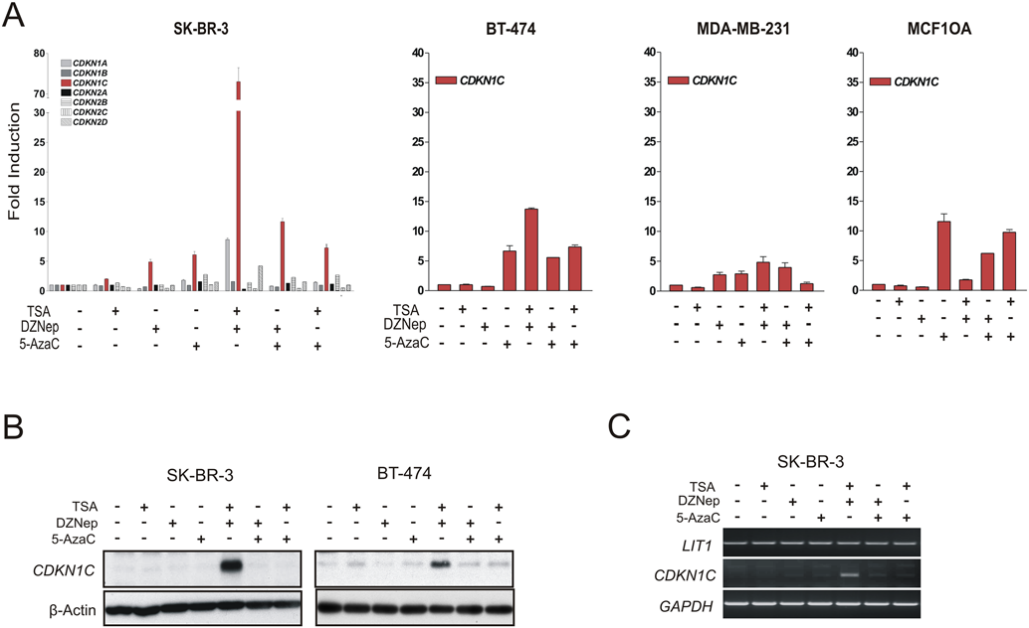
Changes of *CDKN1C* expression in response to DZNep, TSA, Aza and their combinations. (A) SK-BR-3, BT-474, MDA-MB-231 and MCF10A cells were treated with 5 µM DZNep (D), 100 nM TSA (T), and 5 uM 5-AzaC (A) along or in indicated combinations. Expression of *CDKN* members were analyzed by quantitative RT-PCR. Shown were the folds of induction relative to the untreated cells. (B) Western blot analysis of CDKN1C protein expression in SK-BR-3 and BT-474 cells. (C) RT-PCR results show no change of *LIT1* expression upon indicated drug treatment. .

The different chromatin configurations at *CDKN1C* in SK-BR-3, BT-474 and MDA-MB-231 cells may predict differential response of *CDKN1C* expression to various epigenetic drug treatments. We next treated these breast cancer cell lines with DZNep, TSA, or Aza alone or in various combinations and performed quantitative RT-PCR to assess the changes of *CDKN1C* expression upon these treatments. To determine the specificity of the gene response, other CDKI family members were also included for the expression analysis. The results indicate that the combination of DZNep with TSA resulted in a robust induction of *CDKN1C* expression in SK-BR-3 cells, whereas the single drug treatment or other drug combinations, such as DZNep/Aza or TSA/Aza, did not give rise to such a strong induction (Figure 3A). As a marked contrast to *CDKN1C*, other CDKI family members did not respond to a similar extent, revealing the sensitivity of *CDKN1C* to this combination treatment (Figure 3A). Moreover, in BT-474 cells that display a lower abundance of H3K27me3, DZNep/TSA treatment only induced a 12-fold induction of *CDKN1C*, as opposed to a 70-fold induction in SKBR-3 cells. This response was much weaker in MDA-MB-231 cells and MCF10A cells that exhibit low levels of H3K27me3 in *CDKN1C* (Figure 3A). Taken together with the previous results, the data further support the conclusion that H3K27me3 level in *CDKN1C* is closely associated with its expression inversely. It also suggests that inhibition of H3K27me3 by DZNep alone is insufficient but requires TSA treatment for a full reactivation of *CDKN1C*. Lack of response of other *CDKN* family members to DZNep/TSA indicates the lack of similar chromatin state in these genes. Indeed, we found that other CDKIs, such as *CDKN1D*, were not marked by H3K27me3 in SK-BR-3 cells (data not shown). Finally, we confirmed the induction of CDKN1C protein expression in a similar manner by western blot (Figure 3B).

*CDKN1C* is an imprinting gene, whose expression is also negatively regulated by an imprinted control region that contains a non-coding transcript *DMR-LIT1* [30]. To test the possibility that the induction of *CDKN1C* might arise from the downregulation of *DMR-LIT1*, we looked at the expression of *LIT1* upon above drug treatment. The result shows that *LIT1* expression is not changed upon DZNep/TSA treatment (Figure 3C), excluding the possibility that *CDKN1C* induction by DZNep/TSA is the result of *LIT1* downregulation.

### Combination treatment with DZNep and TSA synergistically reverses histone modifications

**Figure 4:**
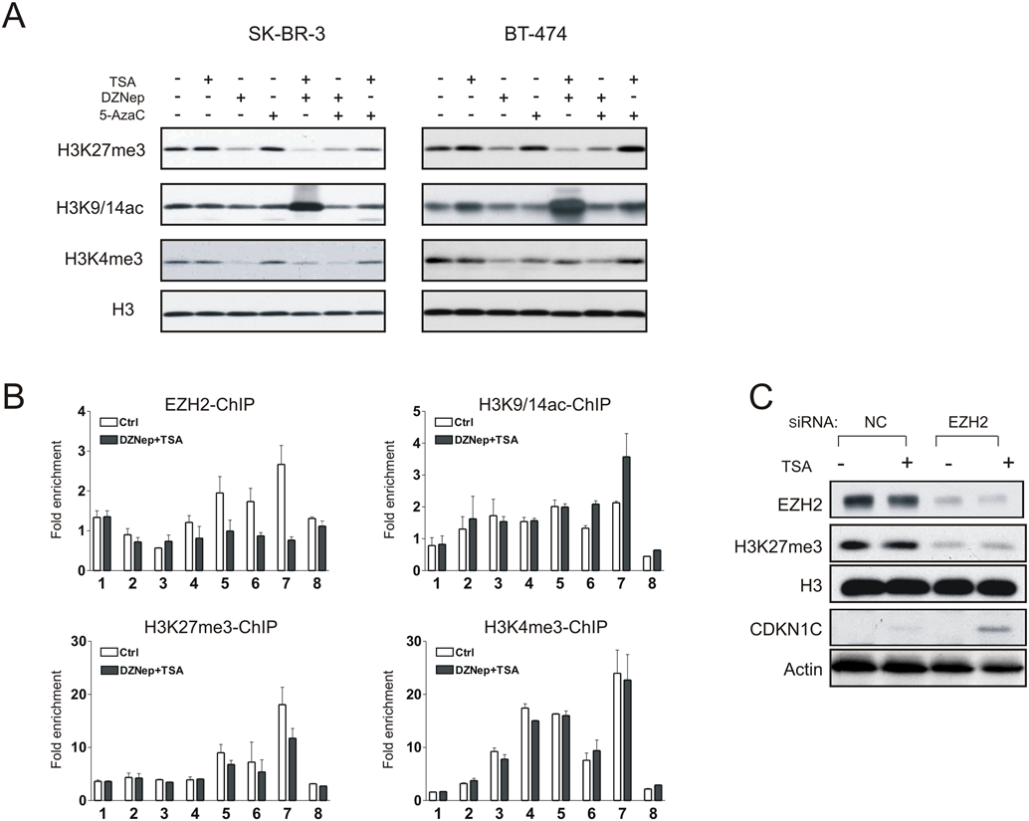
Effects of DZNep/TSA combination on histone modifications. (A) Western blot results show the changes of histone modifications in response to the indicated treatment. Histone proteins were acid-extracted from indicated whole-cell lysates, and indicated histone modifications were analyzed with corresponding antibodies. Histone H3 was used as a loading control. (B) ChIP analysis detects the abundance of EZH2, H3K27me3, H3K4me3 and H3K9/14ac at *CDKN1C* locus before and after the DZNep/TSA combination treatment in SK-BR-3 cells. (C) SK-BR-3 cells were treated with negative control siRNA (NC) or *EZH2* siRNA for 48 h, followed by TSA treatment for 24 h. Changes of CDKN1C, EZH2 and H3K27me3 protein levels were determined by Western blot analysis.

We next examined the bulk histone modifications in cells treated with DZNep, TSA or both. Cellular histone was isolated from cell extracts and subjected to Western blot analysis using antibodies against relevant histone modifications. As shown in Figure 3A, DZNep treatment alone or in combination with TSA caused a diminished H3K27me3 in SK-BR3 and BT-474 cells, as anticipiated from our previous report [23]. A striking observation was the dramatic and synergistic increase in H3K9/14 acetylation in cells treated with the DZNep/TSA combination compared to cells treated with DZNep or TSA single treatment (Figure 4A). Although a decrease in H3K4me3 was also observed in cells treated with DZNep alone, the combination treatment seemed to cause a reverse of this decrease, leading to a level of H3K4me3 comparable to the untreated cells. Thus, the combination of DZNep and TSA induced a reversal of histone modifications by inhibiting H3K27me3 while enhancing acetated H3. This combinatorial effect on histone modification suggests a more permissive chromatin state overall, consistent with the robust induction of *CDKN1C* expression.

We further performed ChIP analysis to determine the changes of chromatin following the above drug treatments. As expected, SK-BR-3 cells treated with DZNep/TSA showed an reduced EZH2 enrichment in *CDKN1C*, with a corresponding decrease in H3K27me3 (Figure 4B). Concomitantly, H3K9/14ac was enhanced after the combination treatment. Thus, taken together with the previous results from quantitative RT-PCR, we identified a strong correlation between the collective changes in histone modifications (H3K27me3 and H3K9/14 ac) and the expression levels of *CDKN1C*. This indicates that combination treatment with DZNep and TSA creates a permissive chromatin environment for *CDKN1C* expression through synergistically reversing associated chromatin marks.

To directly asses the role of EZH2 in *CDKN1C* expression, we used RNA interference to deplete the EZH2 expression. SK-BR-3 cells treated with small interfering RNA (siRNA) targeting EZH2 displayed a marked decrease in EZH2 expression, and a corresponding decrease in bulk H3K27me3 (Figure 4C). Depletion of EZH2, albeit insufficient to induce *CDKN1C* expression, resulted in a marked accumulation of CDKN1C protein in the presence of TSA (Figure 4C). This result is consistent with the previous pharmacology data and further indicates that effective *CDKN1C* induction requires inhibition of both EZH2 and histone deaceylation. It directly supports the model that histone deacetylation and EZH2-mediated histone methylation cooperate to repress *CDKN1C* expression.

### Addition of Aza to DZNep/TSA combination further increases *CDKN1C* expression in BT-474 cells

**Figure 5:**
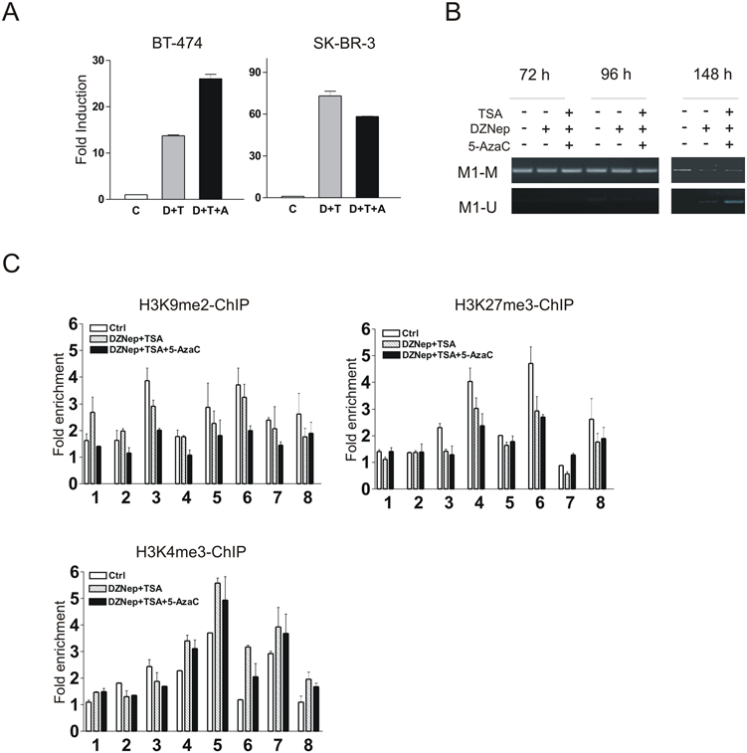
Addition of Aza to DZNep/TSA combination further enhances *CDKN1C* expression in BT-474 cells. (A) Changes of *CDKN1C* expression in BT-474 and SK-BR-3 cells after treatment with DZNep/TSA (D/T) or Aza/DZNep/TSA (D/T/A) were determined by quantitative RT-PCR. (B) MSP analysis of the methylation status of M1 region in BT-474 cells treated with Aza or DZNep/TSA/Aza for indicated times. (C) ChIP analysis showing the changes of H3K9me2, H3K27me3, and H3K9/14ac at *CDKN1C* locus in BT-474 cells untreated and treated with DZNep/TSA or DZNep/TSA/Aza.

In BT-474 cells, the upstream region of *CDKN1C* promoter (M1) was detected to be hypermethylated compared to that in SK-BR-3 cells (Figure 1B). We next examined whether addition of Aza in these cells would further enhance the level of *CDKN1C* induction by DZNep/TSA. BT-474 and SK-BR-3 cells were thus treated with DZNep/TSA in the presence or absence of Aza for 72 h and the changes in *CDKN1C* expression were determined by quantitative RT-PCR analysis. Figure 5A shows the triple treatment in BT-474 cells induced a 27-fold induction of *CDKN1C* expression compared to a 12-fold induction by DZNep/TSA. By contrast, addition of Aza in SB-KR-3 cells did not further increase the level of *CDKN1C* which is already strongly induced by DZNep/TSA. However, MSP analysis revealed that the Aza treatment in BT-474 cells for up to 96 h did not in fact reduce the DNA methylation in the M1 region (Figure 5B), suggesting that the further enhanced induction of *CDKN1C* in BT-474 cells upon the triple combination treatment is not the result of DNA demethylation.

It has been recently reported that Aza can act independently of its ability to inhibit DNA methylation to reactivate gene expression by removing H3K9me2 [31]. Since *CDKN1C* is marked by H3K9me2 in BT-474 cells, we next examined if H3K9me2 in *CDKN1C*, together with other histone marks, have changed after the above treatments. The results show that H3K9me2 level was markedly reduced in cells treated with the three-drug combination, compared with cells treated with DZNep/TSA, while H3K27me3 levels remained the same (Figure 5C). Thus, the further increase in *CDKN1C* expression upon treatment with the triple drug combination in BT-474 cells might be due to additional inhibition of H3K9me2. This is consistent with the fact that Aza treatment did not further increase *CDKN1C* expression in SK-BR-3 in which *CDKN1C* is not marked by H3K9m2. Taken together, we conclude that histone modifications play a predominate role in epigenetic repression of *CDKN1C* in breast cancer cells.

### EZH2-mediated *CDKN1C* repression predicts breast cancer clinical outcome

**Figure 6:**
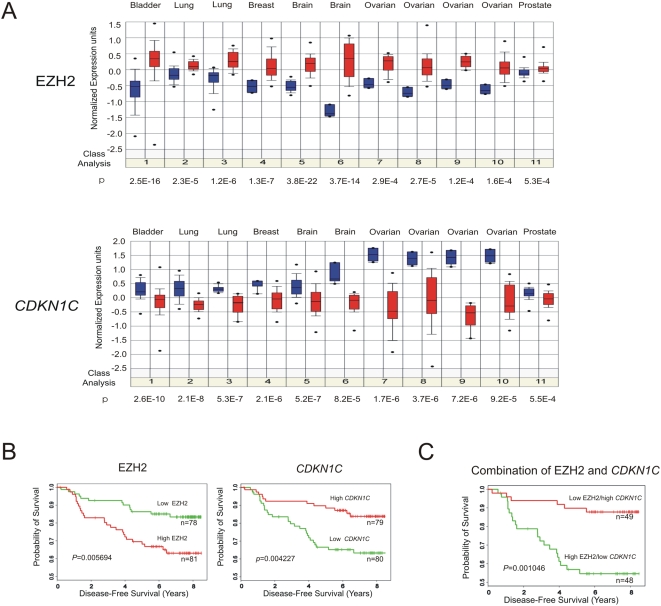
Levels of *CDKN1C* expression predict the clinical outcome of breast cancer patients. (A) Upregulation of *EZH2* and downregulation of *CDKN1C* expression are shown in multiple cancer types with indicated *P* values by comparing the tumor (red) and the adjacent normal (blue) tissues. The data is extracted from Oncomine microarray database (www.oncomine.org). Breast cancer patients were ranked according to different levels of *EZH2* or *CDKN1C* as described in Methods. (B) Kaplan-Meier survival plots of disease-free survival (DFS) from Stockholm cohort (Miller, et. al., GEO ID GSE3494). Patients with higher *EZH2* or C*DKN1C* expression are highlighted in red, while patients with lower *EZH2* or *CDKN1C* expression are highlighted in green. (C) Kaplan-Meier survival plots of disease-free survival (DFS) from Stockholm cohort. Patients with higher *EZH2* but lower C*DKN1C* are highlighted in green, while patients with lower *EZH2* but higher *CDKN1C* are highlighted in red.

Given the involvement of EZH2 in *CDKN1C* repression, we took the advantage of Oncomine microarray database and asked whether their expression levels are reversely correlated in human cancer. The search results revealed that EZH2 is consistently upregulated in multiple human cancer types including breast cancer, while *CDKN1C* is downregulated in these tumors (Figure 6A). This data suggest that EZH2-mediated *CDKN1C* repression might operate widely in human cancers, indicating the potential broad application of pharmacological inhibition of EZH2 for reactivating *CDKN1C* for cancer treatment.

Enhanced EZH2 expression has been previously shown to correlate with poor prognosis of breast cancer [8], [9]. We next determined whether *CDKN1C* as an EZH2-repressed target is reversely associated with the disease outcome. To this end, we examined the publicly available microarray dataset from two breast cancer cohorts with annotated clinical outcome, Uppsala (∼251 patients) and Stockholm (∼159 patients) [32], [33]. The Cox-proportional hazards regression analysis of both disease free survival (DFS) and metastasis-free survival (DMFS) in both data sets showed that *EZH2* and *CDKN1C* are consistently associated with the disease outcome in a reverse manner (Table S1). Specifically, as shown in the Kaplan-Meier plots of Figure 6B, significant poorer outcome in disease free survival (DFS; *P* = 0.004227 and *P* = 0.005694) were observed between Stockholm patients with a higher *EZH2* and a lower *CDKN1C*, respectively. This indicates that both *EZH2* and its target gene *CDKN1C* can be used to predict breast cancer outcome. In comparison, no other *CDKN* family genes could significantly separate the patients by survival (data not shown), consistent with the hypothesis that these genes might not be targeted by EZH2 in breast cancer. Furthermore, we show that breast cancer expressing both higher *EZH2* and lower *CDKN1C* show a much poorer disease-free survival rate (*P* = 0.001046) compared to patients with lower EZH2 and higher *CDKN1C* (Figure 6C). Thus, the combination of both EZH2 and *CDKN1C* may be more predictive of breast cancer recurrence than either one alone. This result suggests that assessing both EZH2 and its target gene may be more accurate to measure the activity of EZH2 pathway. This data also has implications in patient stratification for potential clinical use of EZH2-inhibiting agents such as DZNep. We predict that breast cancer patients with higher EZH2 and lower *CDKN1C* might receive the greatest benefit from cancer therapeutic targeting of EZH2-mediated gene repression.

## Discussion

Our findings suggest that Polycomb protein EZH2-mediated H3K27me3 might be the key chromatin mark associated with the transcriptional repression of *CDKN1C* in breast cancer cells. It also indicates that EZH2 functions through cooperation with histone deacetylation for effective repression of its target genes. Lack of dominate effect of DNA methylation, together with RT-PCR analysis, indicate that *CDKN1C* expression in breast cancer is maintained at low or basal levels rather than completely silenced. Indeed, *CDKN1C* can be only modestly induced by Aza but strongly induced by DZNep/TSA that targets both EZH2-mediated histone methylation and deacetylation. This finding indicates that DZNep/TSA might preferentially target genes whose repression is associated with H3K27me3 but not those completely silenced by DNA methylation. This is consistent with a recent study showing that EZH2 functions to maintain the low expression of target genes that lack DNA methylation but is not required for maintaining gene silencing predominantly caused by DNA methylation [34].

We show that *CDKN1C* carries both repressive (H3K27me3) and activating (H3K4me3) chromatin marks, revealing a “bivalent” chromatin state. Moreover, recent studies have shown that methylation of H3K4 is reversely associated with DNA methylation of gene promoters [35], [36], [37] and that DNMT only recognizes unmethylated H3K4 to induce DNA methylation [38]. Thus, the detection of a strong H3K4me3 in the *CDKN1C* promoter is consistent with the lack of DNA methylation in the vicinity of the *CDKN1C* promoter as we observed in breast cancer cells. A ‘bivalent’ chromatin mark (H3K27me3 and H3K4me3) has been originally described in embryonic stem (ES) cells that is generally associated with genes transcribed in low levels [28], [39]. Recent studies have also indicated its existence in differentiated cells [26], [29]. Many tumor suppressor genes carrying a bivalent chromatin mark in ES cells are subject to further DNA methylation for stable gene silencing in cancer cells [40], [41], [42]. It has been proposed that during this malignant process DNA methylation confers a concomitant loss of H3K4 methylation after a bivalent chromatin is converted to a monovalent state [17], [41], [43]. The retention of the bivalent domain without DNA methylation indicates that this epigenetic mechanism also exists in cancer cells, which might also contribute to the malignant transformation together with the well-characterized DNA methylation. This finding has obvious therapeutic implications. As described above, these bivalent genes that are lowly transcribed (not completely silenced) might be most susceptible to histone modifying compounds such as DZNep/TSA as illustrated in this study, but not to DNA demethylating agents. These genes might contain important tumor suppressors that have been overlooked historically. We therefore speculate that the above described epigenetic treatment might open a new avenue for cancer therapeutics that aim to target this aberrant epigenetic process that has been previously under-appreciated in cancer.

Our comprehensive epigenetic analysis of these observations highlights the emerging concept that multiple epigenetic mechanisms collaborate to repress gene expression in cancer cells. Furthermore, our results indicate that DNA methylation and EZH2-H3K27me3 might not be mechanistically linked as previously suggested [14]. In fact, we did not detect methylated DNA in EZH2-H2K27me3 enriched region in *CDKN1C*. Conversely, in the *CDKN1C* promoter region that appears to be methylated (such as in BT-474 cells), no EZH2-H3K27me3 was detected. This finding is consistent with the recent genomic scale analysis showing that H3K27me3-mediated gene silencing and DNA methylation target different set of genes [15], [17]. Despite the presence of both epigenetic events in the *CDKN1C* locus in BT-474 cells, EZH2-H3K27me3 appears to be the predominate one that is in synergy with histone deacetylation to repress *CDKN1C* expression. Targeting EZH2-H3K27me3 by DZNep would presumably synergize with HDAC inhibitors and/or Aza to maximally restore the tumor suppressor function of *CDKN1C*.

Finally, we show that the downregulation of *CDKN1C* by EZH2 in breast cancer is associated with a poor disease outcome. Mechanistically, this finding is consistent with the previous knowledge that overexpression of EZH2 correlates with a poor breast cancer prognosis. Moreover, a recent report shows that Polycomb repression signature genes can predict clinical outcome of multiple solid tumors [24]. These findings thus suggest the utility of EZH2 target genes as prognostic marks. We further show that the combination of *EZH2* and *CDKN1C* gives a better prediction of disease outcome that achieved through either gene alone. This might suggest that measuring both EZH2 and its target gene activity as the readout might be more accurate in predicting the activity of this silencing pathway. Indeed, our data suggest that EZH2 alone is insufficient but requires other factors such as HDAC to assure a full functionally in repression of certain genes. Therapeutically, this information may provide significant values in patient stratification for potential clinical use of EZH2 inhibitors as anti-cancer agents. Such agents may be particularly useful for patients with breast cancer harboring *EZH2*-mediaed repression of *CDKN1C*. Furthermore, we show that upregulation of *EZH2* and the corresponding downregulation of *CDKN1C* occur in multiple human cancers. This may suggest that the pharmacological approach we have demonstrated for inhibiting EZH2 and reactivating *CDKN1C* might have broad application for cancer therapy.

## Methods

### Cells and drug treatment

Cell lines used in this study were all obtained from the American Type Culture Collection (ATCC). Cells were maintained in appropriate medium conditions until harvested. For drug treatment, cells were treated with 5 µM DZNep (obtained from National Cancer Institute of USA) or 5 µM 5-aza-2′-deoxycytidine (5-AzaC; Sigma) for 72 h, and trichostatin A (TSA; Sigma) at 100 nM for 24 h. For 5-AzaC treatment, the medium was replaced with freshly added 5-AzaC for every 24 h. For co-treatment of cells with 5-DZNep and TSA, DZNep was added for 48 h, and then treated with TSA for additional 24 h.

### RNA interference

The siRNA targeting EZH2 and non-targeting control were purchase from from 1st BASE Pte Led (Singapore) as following sequence: 5′-GACUCUGAAUGCAGUUGCU -3′. SK-BR-3 cells were transfected with 100 nM final concentration of siRNA duplexes using Lipofectamine 2000 (Invitrogen) following the manufacturer's instructions.

### Histone extraction and immunoblot analysis

Whole cell extract was prepared as previously [23]. Histone proteins were acid extracted following Upstate protocol. Western blots were probed with the following antibodies: anti-H3K27me3 (07-449), anti-H3K9me3 (07-442), anti-H3K9/K14ac (06-599), anti-H3K4me3 (07-473), and anti-EZH2 (AC22) were purchased from Upstate. Anti-H3 (3H1) was from Cell Signaling and anti-EZH2 and anti-p57 were from Santa Cruz Biotechnology.

### Quantitative real-time PCR and RT-PCR

Total RNA was isolated from cell lines using Trizol (Invitrogen) and purified with the RNAeasy Mini Kit (Qiagen). Reverse transcription was performed using an RNA Amplification kit (Ambion). Quantitative real-time PCR was performed on a PRISM 7900 Sequence Detection System (Applied Biosystems) using TaqMan probes (Applied Biosystems). Samples were normalized to the levels of GAPDH mRNA. For PCR 100 ng of cDNA was used and the primer sequences are shown in Table S2.

### DNA methylation analysis

The CpG island DNA methylation status was determined by PCR analysis after bisulfited modification (EZ DNA Methylation-Gold Kit, Zymo Research,) and followed by methylation-specific PCR (MSP). Primer sequences are shown in Supplementary Table 2.

### Chromatin Immunoprecipitation (ChIP) assays

ChIP assay was performed as previously with a modified protocol that uses QIAquick PCR purification kit (Qiagen) to purify precipitated DNA. The immunoprecipitapted DNA was quantitated by real-time quantitative PCR using PRISM 7900 Sequence Detection System (Applied Biosystems). Primer sets are designed to amplify approximately 200 bp around the indicated region. The following antibodies were used in this study: anti-H3K27me3 (Upstate), anti-H3K9me3 (Abcam), anti-EZH2 (Upstate, anti-H3K9/K4ac (Upstate), anti-Histone H3(Abcam). Quantification of ChIP results was performed relative to the input amount. The sequences of the PCR primers are shown in Table S2.

### Data set and survival analysis

The breast cancer data set from Uppsala and Stockholm cohorts with relevant clinical information have been described previously [32], [33]. The expression of probes of each *CDKN* gene was averaged and transformed to z-score. The positive z-score was treated as higher expression and the negative z-score was treated lower expression. Using the survival event status and time information, we computed the survival association of expression status (high/low expression) using Cox-Proportional Hazards model implementation (coxph) available in R-library “survival”. Kaplan-Meier survival analysis was used for the analysis of clinical outcome. For the combination of *EZH2* and *CDKN1C*, the average expressions of both *EZH2* and *CDKN1C* genes were separately transformed to z-score. The tumors with opposite signs of z-scores of the *EZH2* and *CDKN1C* were included in the analysis and the tumors with same signs of z-scores were left out of the analysis. Tumors in which *EZH2* up or positive *EZH2* z-score and *CDKN1C* down or negative *CDKN1C* z-score were classified as class with label “0”. Tumors in which *EZH2* down or negative *EZH2* z-score and *CDKN1C* up or positive *CDKN1C* z-score were classified as class with label “1”. Similar survival analysis was carried out using cox proportional hazards model fitting and Kaplan-Meier plots.

## Supporting Information

Table S1 (0.02 MB XLS)

Table S2 (0.03 MB XLS)
