# Correction: *CDKN1C* (p57^KIP2^) Is a Direct Target of EZH2 and Suppressed by Multiple Epigenetic Mechanisms in Breast Cancer Cells

**DOI:** 10.1371/annotation/e70583a2-3581-4848-b1e3-b518ac07d3a6

**Published:** 2009-04-13

**Authors:** Xiaojing Yang, R. K. Murthy Karuturi, Feng Sun, Meiyee Aau, Kun Yu, Rongguang Shao, Lance D. Miller, Patrick Boon Ooi Tan, Qiang Yu

The author contributions are incorrect. The correct author contributions should read as follows: Conceived and designed the experiments: XY RGS QY. Performed the experiments: XY MA, FS. Analyzed the data: XY RKMK PT. Contributed reagents/materials/analysis tools: RKMK FS KY LDM PT. Wrote the paper: XY QY.

